# Our State Medical College

**Published:** 1857-06

**Authors:** 


### Our State Medical College.

We are glad to be able at this early day to place before our readers, in a brief manner, the announcement of this, the Medical Department of the Iowa State University.

It will be remembered that this institution has closed its seventh session, and with all the embarrassments by which it has been surrounded, still continues prosperous, having the last session 86 matriculants with a graduating class of 22. Four years since, the Trustees, in order to disseminate as widely as possible the beneficial influence of the institution, thought proper to establish State scholarships, a limited number of which were lodged in the hands of the principal State officers, to be conferred by them upon such worthy practitioners or students of medicine in their several districts, as might desire the benefits conferred, lessening the cost to the student more than one half. Still desirous of extending the advantages of the State institution, not only to the profession and students of our own State, but to those of the entire west, they have now carried out the original intention and made it a free school. The liberal endowment by the general government to our University, and the sinking fund of the Medical Department, fully justify this liberality.

We therefore say to those wishing to avail themselves of the advantages of this department of our University, that the course of instruction is thorough, meeting the demands of the American Medical Association. And that, while we lessen the cost to the student, we shall exact from the candidate for graduation a thorough knowledge of his profession.

### Medical Faculty.

AMOS DEAN, L. L. D., President of the University.

---

D. L. McGugin, M. D., Professor of Physiology, Pathology, and President of the Medical Faculty.

FREEMAN KNOWLES, M. D., Professor of Theory and Practice of Medicine.

J. C. HUGHES, M. D., Professor of Surgery and Dean of the Faculty.

WELLS R. MARSH, M. D., Professor of Chemistry, Toxicology, and Agricultural Chemistry.

JOHN R. ALLEN, M. D., Emeritus Professor of Obstetrics and Lecturer on Insanity.

A. S. HUDSON, M. D., Professor of Obstetrics and Diseases of Women and Children.

DANIEL MEEKER, M. D., Professor of Anatomy.

MONTGOMERY JOHNS, M. D., Professor of Materia Medica, Therapeutics and Microscopy.

JOHN J. PAGE, M. D., Demonstrator of Anatomy.

A. M. CARPENTER, M. D., Prosector to the Chair of Surgery.

J. WARD DAVIDSON, M. D., Prosector to the Chair of Anatomy.

J. C. HUGHES, M. D., Dean.

It will be seen by the above that A. S. Hudson, M. D one of the original incorporators of the institution, is again with us.

Dr. H. labored arduously for three years during the infancy of the institution, proving himself a thorough teacher, a worthy colleague, and well calculated to advance the interests of any institution with which he may be connected.

At the close of the last session, Feb. 25, 1857, the degree of Doctor of Medicine was conferred by the Presipent of the Faculty, Prof. McGugin, on the following named gentlemen, after which the Valedictory Address was pronounced by Prof. Meeker:

| NAME. | RESIDENCE. | SUBJECT OF THESES. |
|---|---|---|
| Adams, Geo. G. | Pennsylvania, | Variola. |
| Ball, D. K. | Iowa, | Enteric Fever. |
| Coons, Jas. N. | Missouri, | Typhoid Fever. |
| Colings, Isaac S. | Indiana, | Epidemic Dysentery. |
| Elsworth, H. N. | Indiana, | Veratrum Viride in Pneumonia and Typhoid Fever. |
| Fletcher, R. J. | Kentucky, | Pulmonary Affections. |
| Frazer, J. W. | Missouri, | Pneumonitis. |
| Hare, A. J. | Illinois, | Typhoid Fever. |
| Johnson, O. B. | Kentucky, | Dysentery. |
| Kellogg, A. H. | Minnesota Ter'y, | Premature Labor. |
| Leese, Jacob. | Missouri, | Pneumonia. |
| Meeker, Lysander. | Indiana, | Veratrum Viride, its Therapeutic effects in Disease. |
| Nelson, G. W. | Iowa, | Nature in Miasm. |
| Newell, O. W. | Illinois, | Psycology vs. Pathology. |
| Pyle, Eliott. | Iowa, | Diagnosis. |
| Russell, W. A. J. | Illinois, | Pneumonia. |
| Slade, J. W. | Illinois, | Duties of an Accoucher. |
| Taylor, Columbus | Iowa, | Modes of Death. |

**Honorary Degree.**

| | |
|---|---|
| Dr. P. Walker, | Iowa. |
| Dr. George Shedd, | Iowa. |
| Dr. M. W. Vroman, | California. |
| Dr. S. A. Winslow, | Illinois. |

| | |
|---|---|
| Number of Graduates, | 22 |
| Number of Matriculants, | 86 |

